# Poor sleep patterns are associated with the prevalence of benign prostatic hyperplasia in US aged 40 and older: A cross-sectional study based on NHANES

**DOI:** 10.1371/journal.pone.0319434

**Published:** 2025-02-25

**Authors:** Dingliang Zhao, Junjie Han, Chengsen Lv, Jialin Gao

**Affiliations:** 1 Department of Urology II, The First Hospital of Jilin University, Changchun, China; 2 Department of Urology, The Second Hospital of Jilin University, Changchun, China; Trinium Woman’s Hospital, KOREA, REPUBLIC OF

## Abstract

**Background:**

The connection between sleep patterns (sleep duration, trouble sleeping and sleep disorders) and benign prostatic hyperplasia, commonly referred to as BPH, is not yet clear. Our aim is to investigate the impact of sleep patterns on BPH risk in US men aged 40 and older.

**Methods:**

We performed an observational analysis using data from NHANES 2005–2008 on males aged 40 and up, including a total of 2,555 participants. After accounting for confounding variables, we applied weighted multivariable logistic regression to assess the relationship between sleep patterns and BPH risk according to the complex multi-stage sampling design of NHANES.

**Results:**

In this study, 11.79% of the 2,555 American participants aged over 40 reported to have BPH. after adjusting for confounding variables, multivariable logistic regression analysis showed that short sleep duration, compared to healthy sleep duration (7–9 hours), was linked to a significantly higher risk of BPH (OR: 1.92, 95% CI: 1.42–2.41). Trouble sleeping and sleep disorder were also strongly associated with BPH. Moreover, there appears to be a stronger association among those with poor sleep patterns (OR: 2.07, 95% CI: 1.46–2.91).

**Conclusion:**

Poor sleep patterns in men over 40 in the U.S. is significantly linked to a higher incidence of benign prostatic hyperplasia.

## 1. Introduction

Benign prostatic hyperplasia is considered the most frequent urological disorder among elderly men [1]. It is caused by uncontrolled proliferative growth of epithelial and fibromuscular tissue in the transition zone (TZ) and around the urethra [2]. This growth inhibits urine flow via the prostatic urethra, causing lower urinary tract symptoms [3]. Even though BPH is not life-threatening, it can significantly reduce a person’s quality of life [4,5]. It can also result in problems such as urinary tract infections and bladder stones [6].

Sleep is a circadian physiological process that is crucial for maintaining homeostasis and overall health [7,8]. Sleep disturbances can have serious negative impacts on health, including the development of cancer [9]. Increasing evidence suggests that sleep plays a significant role in the development and progression of prostate cancer, particularly in Western countries [10]. Studies have hypothesized that sleep disturbances may influence the development and progression of prostate cancer through various biological mechanisms, including hormonal regulation, immune function, and inflammation. Melatonin is a hormone produced during sleep that is believed to possess anti-cancer properties. Disruptions caused by insufficient sleep may increase the risk of cancer [11]. Additionally, sleep disturbances are associated with increased levels of oxidative stress and inflammation, which may contribute to carcinogenesis [12].

In recent years, the incidence of BPH has been steadily increasing. However, the exact cause remains unclear. However, it may be linked to factors such as inflammatory reaction, aging, hormonal changes, and tissue homeostasis disruption mediated by metabolic syndrome [13–15]. Recent research increasingly indicates that sleep can influence inflammatory reaction, the regulation of hormones like testosterone, and the progression of various diseases [16,17]. Previous studies have indicated a link between poor sleep quality and prostate diseases [18]. Li et al. identified a relationship between sleep and BPH among Chinese men, while a similar link was observed by Kai et al. in Indian men [19,20].

Due to potential differences in lifestyle, healthcare access, and ethnic diversity, there may be variations in the relationship between sleep patterns and the prevalence of BPH. This study aims to explore the relationship between sleep and the occurrence of BPH in American adult men by analyzing data from the United States. This approach will contribute to a more comprehensive understanding of how these factors interact across different population groups. Notably, the findings could contribute to early intervention and preventive measures for BPH in the United States, as well as enhance public awareness of the importance of sleep in relation to BPH. This may promote better sleep hygiene practices.

## 2. Methods

### 2.1. Data origin

In this cross-sectional study, we utilized publicly available data from the National Health and Nutrition Examination Survey (NHANES) for the years 2005–2008. Carried out by the U.S. Centers for Disease Control and Prevention (CDC), NHANES integrates interviews, physical examinations, and laboratory tests to offer crucial data on various health issues, aiding in public health policy and research. For more details on NHANES planning, data collection, and data files, visit http://www.cdc.gov/nchs/nhanes.html. All NHANES procedures are approved by the National Center for Health Statistics (NCHS) Research Ethics Review Board. Participants provided documented consent in accordance with established protocols before participating in the survey. The NCHS Institutional Review Board/ethics review board (IRB/ERB) protocol numbers of 2005–2008 National Health and Nutrition Survey is ‘#2005-06’(the website is https://www.cdc.gov/nchs/nhanes/irba98.htm).

### 2.2. Criteria for participant selection

**Fig 1:**
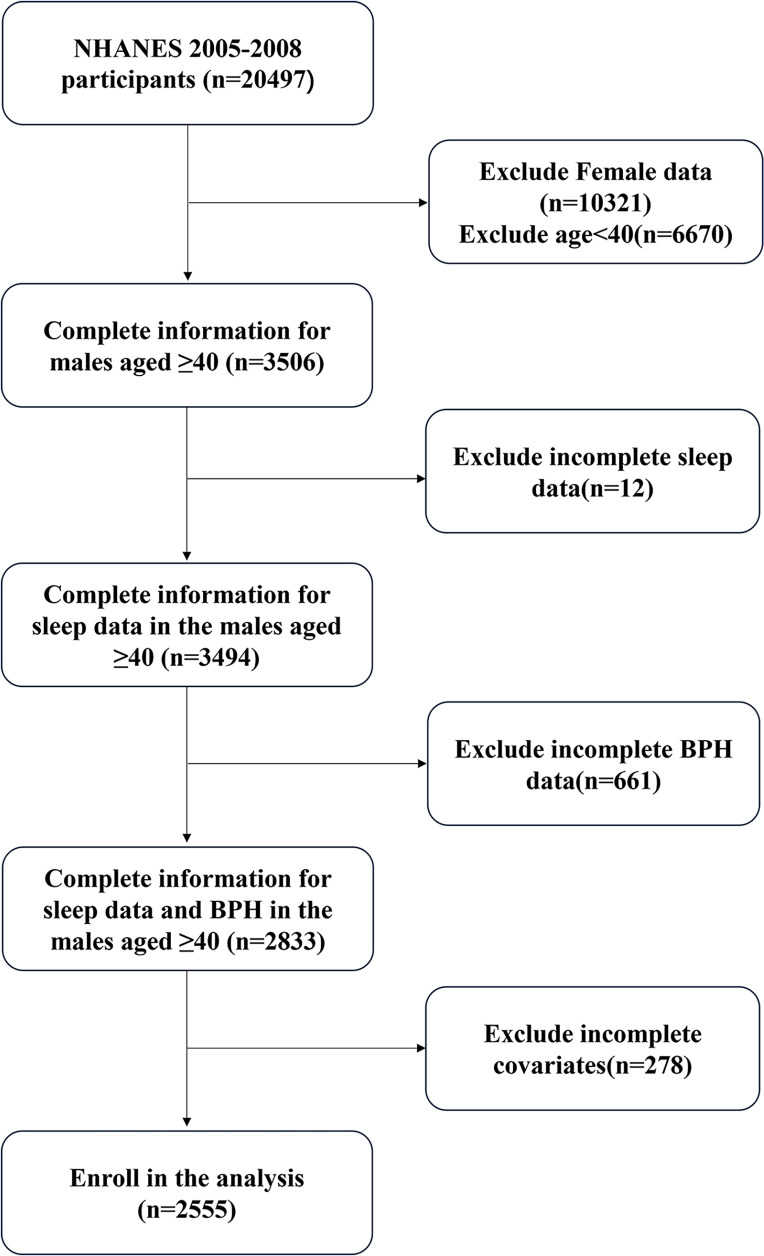
Flowchart of the sample selection from NHANES 2005–2008.

NHANES began collecting data on prostate-related diseases, including BPH and prostate cancer, starting in 2001 and stopped in 2008, while data on sleep disorders started being collected in 2005. This study utilized data from the two NHANES cycles (2005–2008) to explore the association between sleep and the risk of BPH,which included 20,497 participants. We excluded females, individuals under the age of 40, and those with missing data on sleep, benign prostatic hyperplasia, and covariates. This left us with 2,555 participants with complete information. The selection process is shown in Fig 1.

### 2.3. Diagnosis of BPH

All male participants were asked: “Have you ever been told by a doctor or health professional that you have any disease of the prostate? This includes an enlarged prostate.” Those who answered ‘no’ were considered not to have BPH. Participants who answered ‘yes’ were then asked two more questions: ‘Have you been told you have prostate enlargement?’ and ‘Is it benign enlargement?’ Only those who answered ‘yes’ to both questions were classified as having BPH. These questions are represented by the NHANES questionnaire codes “KIQ490”, “KIQ121”, and “KIQ141”. Participants with other prostate conditions, non-benign enlargement, or missing data were excluded.

### 2.4. Assessment of sleep characteristics and description of sleep patterns

Sleep factors were assessed based on three aspects: sleep duration, sleep difficulties, and sleep disorders. During the health interview, self-reported questionnaires (SLD010H) were used to collect data on sleep duration. Each participant was asked to respond to the computer-assisted personal interview question: “How much sleep do you get (hours)?”. Responses were categorized into short sleep duration (<7 hours), healthy sleep duration (7–9 hours), and long sleep duration (>9 hours) [21]. Trouble sleeping were assessed using the specific question (SLQ050): “Ever told doctor had trouble sleeping?”. When participants answer ‘yes’, they are considered to have trouble sleeping. In addition, responses of ‘yes’ or ‘no’ to the question “Ever told by doctor have sleep disorder??” (SLQ060) were used to assess sleep disorders. Each sleep factor was assigned a score based on risk level, where lower-risk factors were given a score of 1 and higher-risk factors a score of 0. The total sleep score, spanning from 0 to 3, was determined by summing these individual scores. A total score of 0 to 1 indicated poor sleep patterns, 2 indicated moderate sleep patterns, and 3 indicated healthy sleep patterns [22].

### 2.5. Assessment of potential covariates

Several covariates were obtained from NHANES demographic, interview, and examination data. These included age, race and family income poverty ratio (≤1, 1–4, ≥4), Education level (less than high school, high school or GED, and more than high school), BMI(≤25, 25–30, and ≥30). Hypertension was identified based on a doctor’s diagnosis of high blood pressure, current use of antihypertensive medication, or having a systolic blood pressure ≥140 mmHg or diastolic blood pressure ≥90 mmHg. Diabetes was defined as being told by a doctor they have diabetes, currently taking antidiabetic medication or insulin, or having a fasting blood glucose level ≥126 mg/dL. Physical activity was assessed based on whether participants engaged in moderate-intensity exercise, fitness, or recreational activities that slightly increased breathing or heart rate for at least 10 minutes at a time each week. Smoking status was categorized as never smokers (smoked < 100 cigarettes in their lifetime), former smokers (smoked > 100 cigarettes but have quit), and current smokers (smoked > 100 cigarettes and are currently smoking). Alcohol consumption was categorized as never drinkers (lifetime consumption < 12 alcoholic drinks), former drinkers (lifetime consumption > 12 drinks but quit), and current drinkers (consumption > 12 drinks per year and currently drinking).

### 2.6. Statistical analysis

Due to the complex multistage sampling design, the appropriate weighting procedures were applied in accordance with NHANES analysis guidelines. Since 2002, NHANES weights have been recalculated every two years. For the 2005–2008 data, the weights cover two 2-year sampling cycles. The recalculated weights are represented as (1/2) ×  WTMEC 2 YR2005-2006 plus (1/2) ×  WTMEC 2 YR2007-2008, where WTMEC 2 YR refers to the NHANES variables for the 2005–2008 cycles. The weighted baseline characteristics of the study population were presented as percentages with 95% confidence intervals (CI), stratified by BPH status. To assess the association between sleep characteristics and patterns with BPH risk, we employed weighted logistic regression models. sleep characteristics included sleep duration, sleep difficulties, and sleep disorders. Model 1 modified for age and race. Model 2 further accounted for education level, poverty-income ratio (PIR), smoking status, and alcohol status. Model 3 included additional adjustments for BMI, hypertension, diabetes, and moderate physical activity. Data analysis for this study was conducted using EmpowerStats (http://www.empowerstats.com).

## 3. Results

### 3.1. Baseline features of participants

There were 2,555 participants in the research. Their characteristics, categorized by self-reported benign prostatic hyperplasia status, are shown in Table 1. The weighted overall prevalence of BPH was 11.79%. Participants with reported BPH were generally older, non-Hispanic White, and had lower educational levels than those without reported BPH. They were also more likely to engage in moderate alcohol consumption and have hypertension (P < 0.05). Additionally, a significantly higher proportion of BPH participants reported experiencing trouble sleeping and sleep disorders.

**Table 1:** Characteristics of participants categorized by reported BPH from NHANES 2005–2008, weighted.

Characteristics	Non-BPH	BPH	P-value
**Age(year)**	(%, 95%*CI*)	(%, 95%*CI*)	<0.0001
40–49	40.71 (36.93, 44.60)	9.78 (5.69, 16.30)	
50–59	32.44 (29.20, 35.86)	23.79 (18.85, 29.55)	
60–69	15.68 (13.73, 17.84)	31.38 (26.75, 36.42)	
70–85	11.18 (9.57, 13.02)	35.05 (29.97, 40.49)	
**Race**			0.001
Mexican American	6.34 (4.79, 8.36)	3.64 (2.05, 6.40)	
Non-Hispanic Black	3.19 (2.09, 4.84)	1.93 (0.86, 4.27)	
Non-Hispanic White	75.86 (71.27, 79.93)	86.52 (80.96, 90.65)	
Other Hispanic	9.75 (7.59, 12.43)	5.22 (2.87, 9.31)	
Other race	4.85 (3.58, 6.55)	2.68 (1.13, 6.25)	
**Education level**			0.2481
Less than high school degree	18.90 (16.28, 21.84)	16.26 (9.34, 26.80)	
High school or GED	26.18 (23.54, 29.00)	21.51 (16.97, 26.88)	
Above high school	54.92 (50.81, 58.96)	62.23 (51.63, 71.77)	
**PIR**			0.1898
≤1	9.42 (7.91, 11.17)	5.71 (3.45, 9.32)	
1–4	46.09 (42.36, 49.87)	47.68 (39.55, 55.94)	
≥4	44.49 (40.33, 48.73)	46.60 (36.50, 56.99)	
**BMI(kg/m** ^**2**^ **)**			0.9502
≤25	21.57 (19.25, 24.10)	22.51 (16.72, 29.60)	
25–30	42.00 (39.08, 44.97)	40.72 (32.24, 49.79)	
≥30	36.43 (33.15, 39.84)	36.77 (29.10, 45.17)	
**Hypertension**			<0.0001
No	53.86 (50.87, 56.81)	37.22 (32.19, 42.53)	
Yes	46.14 (43.19, 49.13)	62.78 (57.47, 67.81)	
**Diabetes**			0.014
No	86.32 (84.48, 87.98)	80.82 (74.72, 85.72)	
Yes	13.68 (12.02, 15.52)	19.18 (14.28, 25.28)	
**Smoking status**			<0.0001
Never	42.85 (39.53, 46.24)	34.18 (25.68, 43.83)	
Former	32.90 (30.48, 35.41)	55.56 (47.33, 63.48)	
Now	24.25 (21.35, 27.39)	10.27 (6.55, 15.74)	
**Alcohol status**			0.6378
Never	5.22 (4.12, 6.60)	6.41 (3.42, 11.71)	
Former	10.99 (8.95, 13.42)	9.97 (7.26, 13.53)	
Now	83.79 (80.93, 86.30)	83.62 (78.54, 87.69)	
**Moderate activity**			0.6289
Yes	49.86 (45.23, 54.50)	51.52 (43.90, 59.07)	
No	50.14 (45.50, 54.77)	48.48 (40.93, 56.10)	
**Sleep time(h)**			0.2741
7–9	60.40 (57.74, 63.00)	54.77 (48.53, 60.87)	
<7	38.37 (35.85, 40.96)	43.49 (37.81, 49.34)	
>9	1.23 (0.79, 1.91)	1.74 (0.65, 4.59)	
**Trouble sleeping**			0.0226
No	76.74 (73.91, 79.35)	70.78 (65.82, 75.28)	
Yes	23.26 (20.65, 26.09)	29.22 (24.72, 34.18)	
**Sleep disorder**			0.0115
No	90.15 (88.36, 91.69)	84.99 (80.01, 88.89)	
Yes	9.85 (8.31, 11.64)	15.01 (11.11, 19.99)	
**Sleep pattern**			0.0149
Healthy	47.41 (44.44, 50.39)	41.67 (36.93, 46.57)	
Intermediate	36.14 (33.05, 39.35)	35.40 (30.35, 40.79)	
Poor	16.45 (14.53, 18.57)	22.93 (18.38, 28.21)	

Categorical variables are described as survey-weighted percentage (95% CI), p-value was by survey-weighted Chi-square test. p <  0.05 presents significant difference. BPH, benign prostatic hyperplasia; h, hour; PIR, Poverty-to-income ratio; BMI, Body mass index; NHANES, National Health and Nutrition Examination Survey.

### 3.2. Relationship between sleep and prevalence of BPH

We employed a multivariable logistic regression analysis to investigate the relationship, as detailed in Table 2. Participants with short sleep duration (<7 hours) had a 1.76-times greater risk of developing BPH, even after controlling for age and race (Model 1). Conversely, those with long sleep duration (>9 hours) did not demonstrate a statistically significant variation in BPH risk compared to those with normal sleep duration. After accounting for additional confounding factors (model 3), the relationship between short sleep duration and BPH remained significant. Furthermore, as compared to regular sleep, both trouble sleeping and sleep disorders were strongly related with a greater chance of developing BPH (trouble sleeping: OR:1.54, 95% CI:1.13–2.10; sleep disorder: OR:1.63, 95% CI:1.07–2.47).

**Table 2:** Association between sleep and risk of BPH.

Sleep factor	Model 1		Model 2		Model 3	
Sleep time(hour)	OR(95%CI)	P-value	OR(95%CI)	P-value	OR(95%CI)	P-value
7–9	ref		ref		ref	
<7	1.76 (1.31, 2.37)	0.001	1.89 (1.40, 2.57)	0.001	1.92 (1.42, 2.41)	0.005
>9	1.06 (0.30, 3.69)	0.932	1.16 (0.33, 4.02)	0.819	1.08 (0.31, 3.77)	0.904
Trouble sleeping						
No	ref		ref		ref	
Yes	1.55 (1.15, 2.08)	0.008	1.55 (1.14, 2.11)	0.013	1.54 (1.13, 2.10)	0.028
Sleep disorder						
No	ref		ref		ref	
Yes	1.70 (1.15, 2.51)	0.013	1.61 (1.06, 2.45)	0.041	1.63 (1.07, 2.47)	0.044

Adjusted for:

Model 1: Age, Race.

Model 2: Model 1, Education level, PIR, Smoking status, Alcohol status.

Model 3: Model 2, BMI, Hypertension, Diabetes, Moderate activity.

Table 3 summarizes the link between sleep patterns and BPH. After accounting for confounding variables (Model 3), persons with poor sleep habits were more likely to have BPH than those with healthy sleep patterns (OR: 2.07, 95% CI: 1.46–2.91).

**Table 3:** Association between pattern sleep and risk of BPH.

Sleep pattern	Model 1		Model 2		Model 3	
	OR (95%CI)	P-value	OR (95%CI)	P-value	OR (95%CI)	P-value
Healthy	ref		ref		ref	
Intermediate	1.51 (1.13, 2.01)	0.010	1.53 (1.15, 2.04)	0.011	1.55 (1.17, 2.05)	0.021
Poor	1.98 (1.44, 2.73)	<0.001	2.06 (1.47, 2.89)	<0.001	2.07 (1.46, 2.91)	0.006
P for trend	<0.001		<0.001		<0.001	

Adjusted for:

Model 1: Age, Race.

Model 2: Model 1, Education level, PIR, Smoking status, Alcohol status.

Model 3: Model 2, BMI, Hypertension, Diabetes, Moderate activity.

### 3.3. Subgroup analysis

A further subgroup analysis was done to examine the reliability of the relationship, as shown in S1 Table. This analysis looked at the potential impact of age, education Level, PIR, BMI, hypertension, and diabetes on the observed relationship. Our results showed that none of these factors were statistically significant, suggesting they did not meaningful affect the observed relationship (all P for interaction >  0.05).

## 4. Discussion

In this research, we included a nationally representative sample of men aged 40 and older to investigate the link between sleep and BPH. Our analysis revealed several key findings. First, after controlling for confounding variables, individuals with shorter sleep durations had a considerably increased chance of getting BPH than those with normal sleep durations. Conversely, long sleep durations did not show a statistically significant impact on BPH risk. Moreover, those experiencing trouble sleeping or sleep disorders had an elevated risk of BPH. Second, poor sleep patterns were linked to a greater risk of BPH compared to healthy sleep patterns. Finally, subgroup analyses demonstrated that age, Education Level, PIR, BMI, hypertension, and diabetes did not significantly alter this association, thus reinforcing the robustness of our results.

Our findings align with those of a cross-sectional study from India, which also reported a robust link between poor sleep quality and BPH [20]. However, this study was based on an Indian sample and explored the link using sleep quality scores. Unlike the Indian study, our research focuses on a U.S. population sample and investigates the relationship between BPH and three distinct sleep features: sleep duration, sleep difficulties and disorders. This offers a broader perspective and adds valuable insights to cross-sectional studies on this topic within the American context. Another finding from China found that shorter sleep duration is strongly associated with an elevated incidence of lower urinary tract symptoms (LUTS)/BPH in middle-aged and older men [19]. Moreover, a Mendelian research indicated that sufficient sleep duration could act as a protective factor against BPH, supporting a causal relationship between sleep levels and BPH. The study utilized data from the FinnGen Consortium database, with BPH cases identified based on ICD coding system standards [23].

Although there is a link between sleep and BPH, the underlying mechanism remains unclear. Some research suggests that sleep, a crucial part of the circadian rhythm, may affect BPH by influencing this cycle [24]. The circadian rhythm influences the body in various ways. On the one hand, disrupting the circadian rhythm can increase inflammation and make the immune response stronger [25]. Inflammatory responses can cause chronic tissue damage to the prostate. As the injury heals repeatedly, the prostate tissue can become enlarged due to continued hyperplasia [26]. On the other hand, changes in the circadian rhythm can affect the development of metabolic syndrome [27–29]. Furthermore, metabolic syndrome is a significant trigger for BPH development [30]. Additionally, sleep can affect the body’s endocrine system [31]. Androgens, particularly testosterone, are key factors in the development of BPH [32]. Research shows that sleep duration and continuity significantly affect hormone level fluctuations, especially testosterone. This makes androgen-dependent benign prostatic hyperplasia more likely to occur [33,34].

Despite it was a nationwide study, there are some limitations. First, the study used self-reported BPH diagnoses instead of objective tests like prostate ultrasound. This could affect the results. Second, even though we adjusted for major confounders, residual confounding may still exist due to the observational nature of the study. Third, symptom of BPH can lead to sleep disruptions, particularly in the elderly population. So, insufficient data about the sequence between the diagnosis of BPH and the occurrence of bad sleep patterns limited our further accurate analysis. Lastly, as a cross-sectional study, it may not fully elucidate the causal association between sleep patterns and BPH. Further prospective design is necessary to validate these findings.

## 5. Conclusion

Our studies show that poor sleep patterns are linked to an increased prevalence of BPH among males aged 40 and older in the United States. However, further future design is needed to better understand this connection.

## Supporting information

S1 Table The subgroup analysis and interactive effect based on sleep pattern. (DOCX)

## Abbreviations

NHANES: National Health and Nutrition Examination Survey
BPH: Benign prostatic hyperplasia
PIR: Poverty-to-income ratio
BMI: Body mass index.
