# Supplementary material for: Poor sleep patterns are associated with the prevalence of benign prostatic hyperplasia in US aged 40 and older: A cross-sectional study based on NHANES
Source: PLoS One. 2025 Feb 25;20(2):e0319434. doi: 10.1371/journal.pone.0319434 (PMC11856457; doi:10.1371/journal.pone.0319434)
Supplement: S1 Table — (DOCX) [file pone.0319434.s001.docx]

**S1 Table :The subgroup analysis and interactive effect based on sleep pattern.**

| Character | Healthy | Intermediate | Bad | P for trend | P for interaction |
| --- | --- | --- | --- | --- | --- |
| Age (%, SE) |  |  |  |  | 0.77 |
| 40-49 | Reference | 1.25(0.28,5.60) | 1.54(0.44,5.42) | 0.48 |  |
| 50-59 | Reference | 2.60(0.97,7.00) | 3.80(1.32,10.92)* | 0.02 |  |
| 60-69 | Reference | 1.55(0.92,2.62) | 1.48(0.65,3.36) | 0.18 |  |
| 70-85 | Reference | 1.28(0.76,2.16) | 1.89(0.92,3.87) | 0.11 |  |
| Education Level  (%, SE) |  |  |  |  | 0.19 |
| Less than high school degree | Reference | 1.55(0.73,3.28) | 1.06(0.45,2.47) | 0.48 |  |
| High school or GED | Reference | 2.04(0.84, 4.93) | 4.57(1.44, 14.49) | 0.03 |  |
| Above high school | Reference | 1.42(0.94,2.15) | 1.74(1.12,2.70)* | 0.35 |  |
| PIR(%,SE) |  |  |  |  | 0.55 |
| ≤1 | Reference | 1.29(0.51, 3.26) | 1.09(0.22, 5.36) | 0.65 |  |
| 1-4 | Reference | 1.40(0.85, 2.31) | 1.97(1.08, 3.61)* | 0.01 |  |
| ≥ 4 | Reference | 1.70(0.94, 3.06) | 2.45(1.15, 5.23)* | 0.02 |  |
| BMI (%, SE) |  |  |  |  | 0.43 |
| ≤25 | Reference | 1.76(0.72, 4.32) | 2.95(0.86, 10.10) | 0.06 |  |
| 25-30 | Reference | 1.60(0.97, 2.66) | 2.72(1.26, 5.86)* | <0.001 |  |
| ≥30 | Reference | 1.32(0.79, 2.20) | 1.47(0.85, 2.54) | 0.10 |  |
| Hypertension (%, SE) |  |  |  |  | 0.15 |
| No | Reference | 2.54(1.41, 4.56)** | 3.48(1.48, 8.16)** | 0.005 |  |
| Yes | Reference | 1.11(0.74, 1.67) | 1.56(0.94, 2.61) | 0.03 |  |
| Diabetes (%, SE) |  |  |  |  | 0.80 |
| No | Reference | 1.45(0.94, 2.22) | 2.10(1.36, 3.24)** | 0.002 |  |
| Yes | Reference | 2.22(1.17, 4.22)* | 1.34(0.47, 3.85) | 0.16 |  |

**p < 0.01 and *p <0.05.
